# Perceptions of Artificial Intelligence in Medical Documentation: A Cross-Sectional Study of Healthcare Professionals

**DOI:** 10.7759/cureus.96604

**Published:** 2025-11-11

**Authors:** Uday Mahajan, Salman Shoukat Ali Parpia, Ria Gupta, Edward Spurrier, Meraj Akhtar, Vibhore Gupta

**Affiliations:** 1 Trauma and Orthopaedics, Queen Elizabeth Hospital Birmingham, Birmingham, GBR; 2 Accident and Emergency, Queen Elizabeth Hospital Birmingham, Birmingham, GBR; 3 Emergency Medicine, University of Birmingham, Birmingham, GBR; 4 Trauma and Orthopedic, Nottingham University Hospitals National Health Service (NHS) Trust, Nottingham, GBR; 5 Emergency Department, Queen Elizabeth Hospital Birmingham, Birmingham, GBR

**Keywords:** ai adoption, artificial intelligence in medicine, clinical governance, electronic health records, survey methodology

## Abstract

Background: Artificial intelligence (AI) is increasingly used across healthcare, yet its role in everyday medical documentation remains uncertain. This study explored the current use, perceptions, and readiness for AI integration among healthcare professionals within a tertiary trauma unit in the United Kingdom.

Methods: A cross-sectional survey was conducted between August and September 2025 using Microsoft Forms (Redmond, USA). The questionnaire assessed AI familiarity, current and intended use, perceived safety and ethics, and awareness of institutional policies. Quantitative and qualitative responses were analysed descriptively.

Results: Sixty-three healthcare professionals completed the survey, including consultants (n = 3, 5%), registrars (n = 12, 19%), core trainees (n = 16, 25%), foundation doctors (n = 6, 10%), and allied health professionals (n = 24, 38%). Familiarity with AI tools was highest among registrars and core trainees and lowest among consultants. While only 52% (n = 33) had used AI for clinical or administrative work, 70% (n = 44) indicated they would do so if officially approved. Participants perceived AI as potentially useful but raised concerns about data security (n = 47, 75%), clinical inaccuracy (n = 40, 63%), and accountability (n = 38, 60%). Institutional guidance was rare (n = 7, 11%), though most respondents supported formal training (n = 43, 68%) and regulation (n = 54, 86%).

Conclusion: Clinicians across all grades recognise AI’s potential to improve documentation efficiency but remain cautious about safety and governance. Adoption is currently driven by junior and mid-career clinicians, highlighting the need for structured education, clear policies, and responsible oversight to support safe and ethical AI integration into clinical workflows.

## Introduction

Clinical documentation is an essential component of modern healthcare, ensuring continuity of care, communication between professionals, and compliance with legal and governance standards [1]. However, increasing administrative and regulatory demands have significantly expanded the documentation burden on clinicians. Studies suggest that doctors spend between a quarter and half of their working time on clerical tasks, often at the expense of direct patient contact [2]. This administrative workload contributes to stress, burnout, and inefficiency across healthcare systems [2].

Artificial intelligence (AI), particularly large language models (LLMs) such as ChatGPT (California, United States) and Microsoft Copilot (Redmond, USA), has been proposed as a potential solution to alleviate this burden [3]. These tools can assist in generating clinic letters, summarising patient interactions, drafting appraisals, and supporting academic writing. Early research and pilot studies indicate that AI-assisted documentation can improve workflow efficiency and reduce time spent on repetitive tasks [4]. However, the use of AI in clinical documentation remains in its infancy, with wide variation in adoption and limited evidence on real-world use by healthcare professionals [5].

Concerns have been raised regarding the accuracy, safety, and ethical implications of AI-generated text in medical practice. Issues such as factual errors, data privacy, accountability, and potential over-reliance on technology remain significant barriers to integration. At the same time, healthcare institutions have been slow to establish governance frameworks or provide formal training to guide responsible AI use [6,7].

While several studies and systematic reviews have examined the technical performance of AI and large language models in healthcare, few have investigated how clinicians themselves are engaging with these tools in routine documentation and communication [8]. This study addresses that practical gap by exploring user-level familiarity, perceptions, and governance needs surrounding AI use in everyday clinical and academic practice.

## Materials and methods

This study employed a cross-sectional survey design to evaluate healthcare professionals’ perceptions and use of artificial intelligence in medical documentation. The survey was designed by the authors following a review of existing literature on AI-assisted documentation, administrative workload in healthcare, and the ethical considerations of AI use. Draft questions were piloted among a small group of clinicians to ensure clarity and relevance before final dissemination.

The final questionnaire consisted of five sections: demographics; familiarity with AI tools; use of AI in workplace documentation; use of AI in personal or academic activities; and views on safety, ethics, and institutional governance. Most questions were multiple-choice or five-point Likert scale items, with open-text boxes provided for optional qualitative comments.

The survey was distributed electronically via Microsoft Forms between 1 August 2025 and 30 September 2025. It was circulated among clinical staff working in a tertiary trauma unit in the United Kingdom, including doctors, allied health professionals, and other healthcare workers. Participation was voluntary and anonymous. No identifiable data were collected.

Quantitative data were analysed descriptively using Microsoft Excel (Redmond, USA), with frequencies and percentages used to summarise categorical variables. Free-text responses were examined using thematic analysis to identify common themes and representative comments.

Formal ethical approval was not required, as the study involved anonymous responses from healthcare professionals and did not include patient data. The survey complied with data protection and confidentiality standards in accordance with the General Data Protection Regulation (GDPR).

## Results

A total of 63 healthcare professionals completed the survey, representing a range of clinical backgrounds across a tertiary trauma unit in the United Kingdom. After reclassification of job titles, respondents included consultants (n = 3, 5%), registrars (n = 12, 19%), core trainees (n = 16, 25%), foundation doctors (n = 6, 10%), and allied health professionals (n = 24, 38%). Most respondents (n = 48, 76%) had less than 10 years of experience (Table 1).

**Table 1:** Participant characteristics and AI familiarity (n = 63)

Variable	Category	n (%)
Role	Consultant	3 (5)
	Registrar (ST3+)	12 (19)
	Core Trainee (CT1–2 / IMT)	16 (25)
	Foundation Doctor (FY1–FY2)	6 (10)
	Allied Health Professional	24 (38)
Years in Practice	< 10 years	48 (76)
	≥ 10 years	15 (24)
Familiarity with AI Tools	Not familiar / never used	24 (38)
	Occasionally use	25 (40)
	Frequently / heavily use	14 (22)

**Figure 1:**
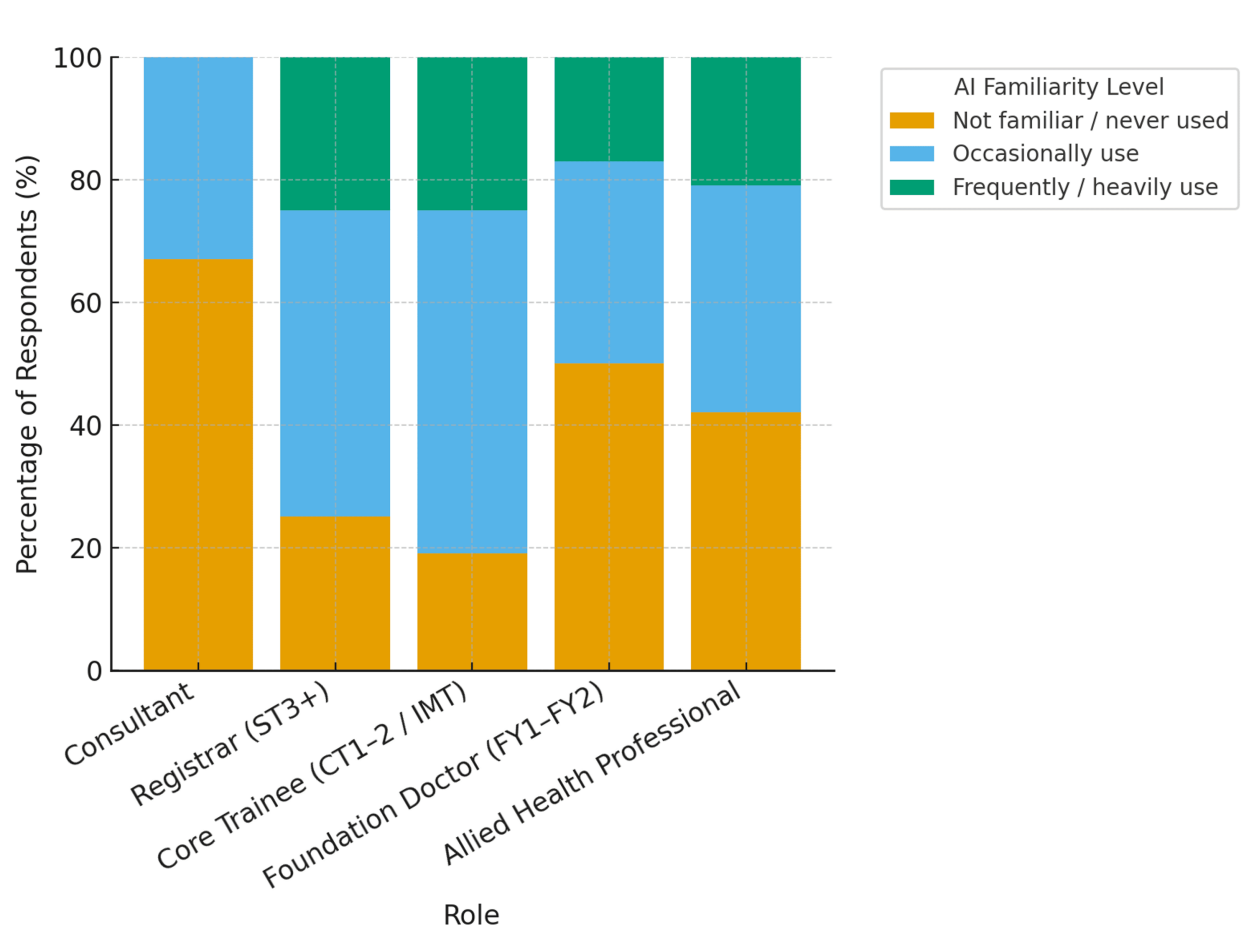
AI familiarity by role

Familiarity with artificial intelligence (AI) tools varied: 24 (38%) were not familiar or had never used them, 25 (40%) occasionally used them, and 14 (22%) reported frequent or heavy use (Figure 1). Registrars and core trainees were the most experienced users, while consultants and foundation doctors showed lower familiarity. Those with fewer than 10 years in practice (n = 48, 76%) were notably more likely to use AI, suggesting greater adoption among early- and mid-career clinicians.

AI use was reported primarily for work-related emails (n = 19, 30%), feedback (n = 19, 30%), appraisals (n = 16, 25%), and incident documentation (n = 13, 21%). Nearly half (n = 30, 48%) had not yet used AI in their workplace, although more than half stated they would use it for note writing (n = 40, 63%), appraisals (n = 34, 54%), and correspondence (n = 33, 52%) if officially approved. Personal and academic use was more common, especially for CV writing, editing, and revision planning (n = 31, 49% each), with one-third (n = 21, 33%) using AI for research or presentation preparation.

Perceptions of AI were generally positive but cautious. Forty-three percent (n = 27) considered AI safe or mostly safe, 51% (n = 32) were uncertain, and 6% (n = 4) viewed it as unsafe. The most frequent concerns were data privacy (n = 47, 75%), inaccuracy (n = 40, 63%), legal accountability (n = 38, 60%), and loss of writing skills (n = 31, 49%). Institutional governance was limited, with only seven respondents (11%) aware of any policy. Nonetheless, a large majority supported developing formal guidance (n = 54, 86%) and wanted structured training on responsible AI use (n = 43, 68%).

Qualitative comments reflected optimism about AI’s potential to reduce administrative workload and improve efficiency, particularly through automated documentation and voice-to-text transcription. However, several participants highlighted the need for robust governance, data protection, and safeguards against over-reliance or professional deskilling.

## Discussion

This study explored healthcare professionals’ use and perceptions of artificial intelligence (AI) in medical documentation within a tertiary trauma unit in the United Kingdom. The findings demonstrate cautious optimism toward AI, with many respondents recognising its potential to reduce administrative workload and improve efficiency, yet expressing persistent concerns regarding safety, accuracy, and governance.

Familiarity and use of AI tools varied considerably across professional groups. Registrars and core trainees were the most active users, while consultants and foundation doctors were the least familiar. This pattern suggests that AI adoption is currently driven by early- and mid-career clinicians, reflecting greater digital literacy and exposure to new technologies during training. Similar trends have been reported in previous studies, where younger clinicians exhibit higher confidence in using digital tools for communication and documentation [9,10]. Senior clinicians, in contrast, often adopt a more cautious approach, emphasising the need for oversight and accountability in AI-assisted clinical practice.

Although most participants had experimented with AI in personal or academic contexts, workplace integration remained limited. The sharp rise in intended use across nearly all documentation tasks if AI were formally approved suggests that regulatory uncertainty, rather than lack of interest, is the main barrier to adoption. This aligns with emerging literature indicating that the absence of clear institutional guidance and medico-legal frameworks is a major limiting factor for clinical AI implementation [11-13].

Safety and ethical concerns were prominent, particularly around data privacy, clinical inaccuracy, and professional accountability. These findings mirror those from wider surveys of healthcare workers, where uncertainty about AI’s reliability and the potential for “hallucination” of false information undermine trust [14]. Importantly, participants in this study balanced these concerns with a strong desire for structured governance and formal training, indicating readiness for responsible adoption once adequate safeguards are in place.

Qualitative comments emphasised a practical, workload-driven interest in AI applications such as automated documentation, voice-to-text transcription, and triage assistance. These areas align with existing NHS digital priorities aimed at reducing clerical burden and improving workflow efficiency [15,16]. However, participants also warned against excessive reliance on AI-generated text, highlighting risks to professional writing standards and the importance of maintaining clinician authorship. This includes developing robust ethical frameworks and ensuring data privacy, given that these factors are critical for fostering trust and wider acceptance among healthcare professionals [17]. These interpretations reflect participants’ perceptions and anticipated benefits rather than measured outcomes and should be understood within the context of self-reported opinions.

The findings underscore the need for clear institutional policy, standardised training, and transparent accountability frameworks before widespread AI deployment in clinical documentation. Tailored education programmes should target different experience levels, combining digital literacy training for senior staff with guidance on data protection and ethical use for all users. 

This study has some limitations. It was conducted within a single tertiary trauma unit, which may limit generalisability to other healthcare settings and specialties. The sample size was modest and relied on voluntary participation, introducing the possibility of selection bias, as individuals with an existing interest in AI may have been more likely to respond. The survey design captured self-reported attitudes and familiarity, which may not accurately reflect actual usage behaviour. Future multi-centre, prospective studies with larger and more diverse samples are warranted to confirm these findings and explore differences across institutions. Additionally, the study’s cross-sectional nature prevents assessment of changes in perception or adoption over time. Despite these limitations, the findings provide valuable insight into real-world perspectives on AI use in clinical documentation and highlight key areas for governance and training development.

## Conclusions

Healthcare professionals view artificial intelligence as a promising tool to improve efficiency and reduce administrative workload in medical documentation. However, these perceptions represent anticipated benefits rather than directly observed effects, underscoring the need for prospective research to evaluate AI’s real-world impact on clinical workflows. The clear gradient in familiarity between junior and senior staff suggests that successful integration will require tailored education and robust institutional governance. Establishing clear policies, training programmes, and oversight frameworks is essential to ensure that AI is implemented safely, ethically, and effectively across clinical settings.

## Appendices

AI use in clinical and academic medical practice

This survey explores the growing use of AI tools (e.g., ChatGPT, Copilot, Grammarly) across

clinical documentation, portfolio, appraisal, and academic tasks. We're interested in your

views on its usefulness, risks, and workplace policies. Responses are anonymous.

Section 1: About You

1. What is your role?

- Consultant

- Registrar (ST3+)

- Core Trainee (CT1-2 / IMT)

- Foundation Doctor

- Allied Health Professional

- Other (please specify)

2. What is your specialty? ____________________

3. How many years have you been practicing medicine?

- 0-2

- 3-5

- 6-10

- 11+

4. How familiar are you with AI tools (e.g., ChatGPT, Copilot, Grammarly)?

- Not familiar

- Aware but never used

- Occasionally use

- Frequently use

- Heavily rely on them

Section 2: Use of AI for Institutional Tasks

5. Which of the following workplace-related tasks have you used AI for?

- Writing clinic letters

- Drafting patient notes

- Composing work-related emails

- Writing or receiving feedback

- Writing appraisals

- Preparing ARCP documents

- Documenting complaints/incidents

- None

- Other (please specify)

6. How frequently do you use AI for these tasks?

- Never- Rarely

- Sometimes

- Often

- Always

7. How useful do you think AI could be for the following institutional tasks, if it were

officially approved and encouraged in your workplace? (1 = Not useful → 5 = Extremely

useful)

- Clinic letters

- Patient notes

- Work emails

- Appraisals

- Feedback

- ARCP documents

8. If AI tools were formally approved for clinical documentation, how convenient or time-

saving do you think their use would be in your day-to-day practice?

- Very convenient

- Somewhat convenient

- Neutral

- Somewhat inconvenient

- Very inconvenient

Section 3: Use of AI for personal Tasks

9. Which personal or academic activities have you used AI tools for?

- Research assistance

- CV writing/editing

- Portfolio reflections

- Studying or revision planning

- Preparing teaching materials

- Writing presentations or abstracts

- General writing

- Other (please specify)

10. How frequently do you use AI for these tasks?

- Never

- Rarely

- Sometimes

- Often

- Always

11. How useful do you find AI in these academic/personal contexts? (1 = Not useful → 5 =

Extremely useful)Section 4: Safety, Ethics & Governance

12. Do you believe AI-generated content in medical practice is:

- Entirely ethical

- Mostly ethical

- Depends on the context

- Somewhat unethical

- Unethical

13. Do you consider current AI use in your setting to be:

- Safe

- Mostly safe

- Unclear

- Potentially unsafe

- Unsafe

14. What concerns (if any) do you have about AI use in medicine?

- Patient data privacy

- Legal responsibility

- Clinical inaccuracy

- Loss of clinical writing skills

- Institutional reputation

- No major concerns

- Other (please specify)

15. Should AI-generated text be clearly disclosed in clinical or academic documents?

- Yes always

- Only in patient-facing documents

- No

- Unsure

Section 5: Workplace Policies & Future Needs

16. Does your current employer have any guidance or rules about the use of AI tools in

documentation?

- Yes - written policy

- Verbal advice only

- Not sure

- No policy at all

17. Have you ever received formal training or guidance on the responsible use of AI tools in

your work?

- Yes

- No

18. Would you support official NHS or institutional guidance on acceptable AI use?

- Strongly support- Support

- Neutral

- Oppose

- Strongly oppose

19. Would you benefit from AI training focused on clinical or academic writing?

- Yes

- No

- Maybe

Final Comments

20. Please share any experiences, opinions, or ideas about AI in your medical practice:

____________________________________________________________________

____________________________________________________________________

___________________________________________
